# Supplementary material for: Noumeavirus replication relies on a transient remote control of the host nucleus
Source: Nat Commun. 2017 Apr 21;8:15087. doi: 10.1038/ncomms15087 (PMC5413956; doi:10.1038/ncomms15087)
Supplement: Supplementary Information — Supplementary Figures, Supplementary Tables, Supplementary Note, Supplementary Methods and Supplementary References [file ncomms15087-s1.pdf]

Supplementary Figures

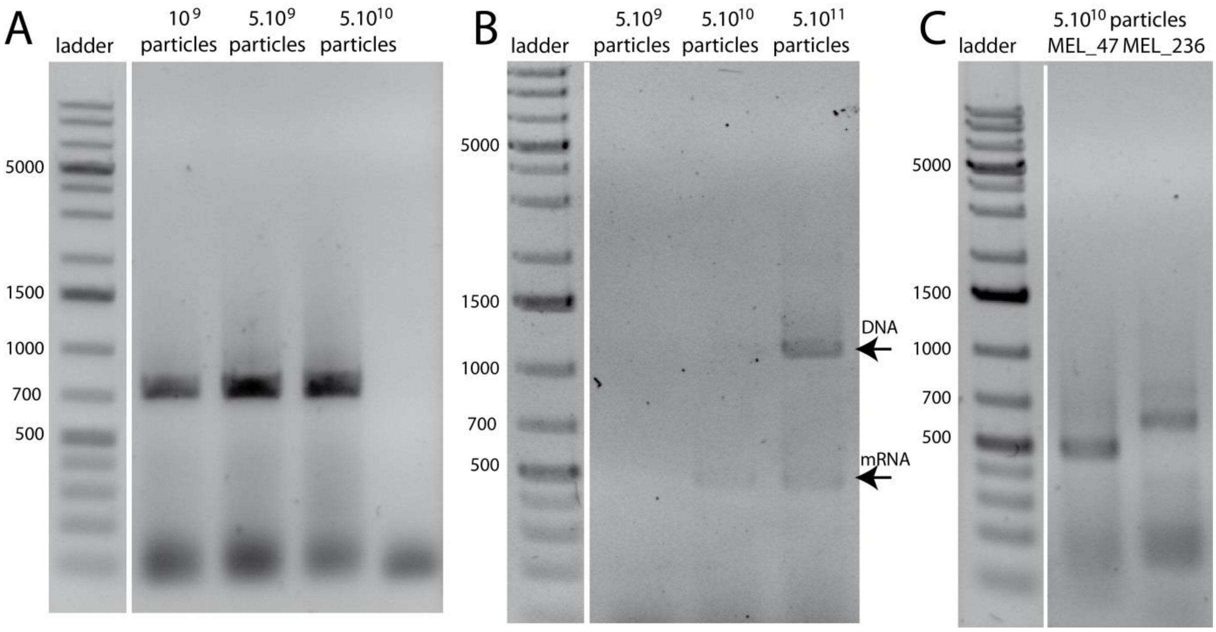

Supplementary Figure 1: PCR gels

A) PCR product of the reverse transcribed GFP mRNA added to Melbournevirus virions extracts at the level of 1 molecule of mRNA per virion. The last lane corresponds to the not reverse transcribed GFP mRNA used as a negative control. B) PCR products of the reverse transcribed RPB1 (MEL_47) mRNA after RNA extraction and mRNA purification. The PCR product is only visible when using >5.10^{9} viral particles. For 5.10^{11} particles, the PCR product of the RPB1 DNA is more intense than the one of the mRNA. C) PCR products of the Melbournevirus reverse transcribed RPB1 and of the most abundant protein (MEL236). No amplicon was obtained with primers specific for the cellular RPB1 or PolyA polymerase.

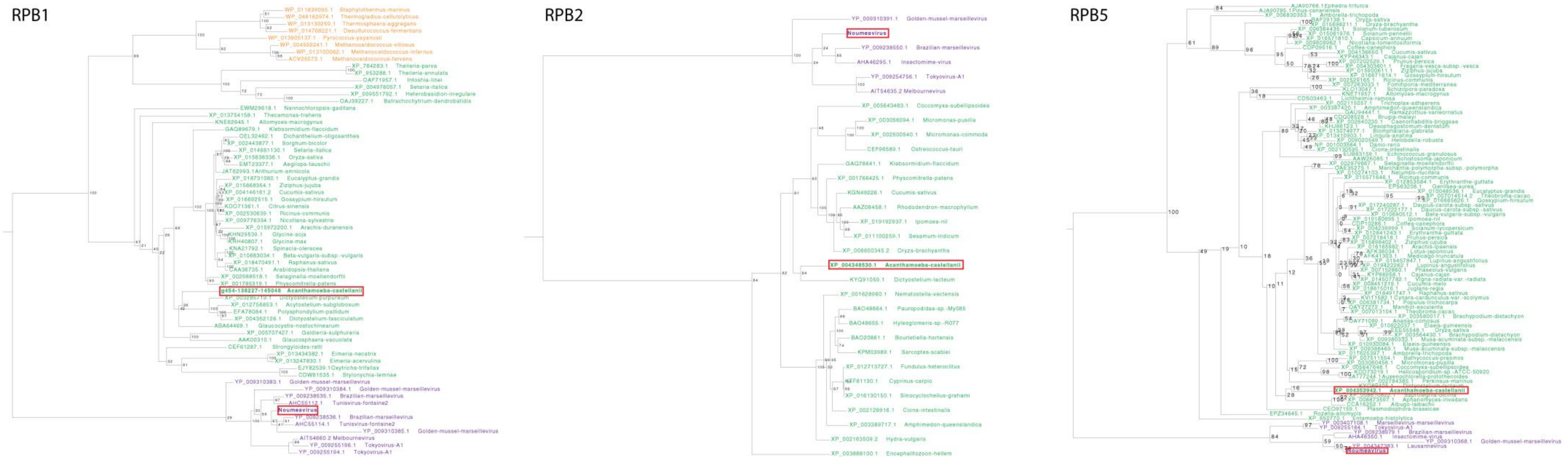

Supplementary Figure 2: Phylogenetic analysis of Noumeavirus transcription-related genes: Maximum likelihood phylogenetic trees of three subunits of the RNA polymerase (RPB1, RPB2 and RPB5) using RaxML[1]. Eukaryotes are shown in green, archaea in orange and viruses in purple. Noumeavirus and *A. castellanii* proteins are indicated by red boxes. Numbers at the nodes indicate bootstrap values. The presented phylogeny does not support recent horizontal gene transfer of the transcription genes from the host to the Marseilleviridae genomes, but does not rule out possible ancient transfers that would support the accretion theory for viruses' evolution (see for review[2]).

Supplementary Tables

Supplementary Table 1 : Comparison of Noumeavirus and Melbournevirus virion proteomes

Origin	Protein Ids NMV	Protein name	Equivalent MEL	Final rank in NMV	Final rank MEL
Noumeavirus	NMV_189	hypothetical protein	MEL_236	1	1
Noumeavirus	NMV_078	histone H3-like protein	MEL_368	2	3
Noumeavirus	NMV_116	major capsid protein	MEL_305	3	4
Noumeavirus	NMV_149	hypothetical protein	MEL_274	4	5
Noumeavirus	NMV_171	Papain-likecysteine protease	MEL_369	5	6
Noumeavirus	NMV_079	histone H2B/H2A fusion protein	MEL_247	6	7
Noumeavirus	NMV_185	hypothetical protein	MEL_241	7	10
Noumeavirus	NMV_333	hypothetical protein	MEL_089	8	39
Noumeavirus	NMV_141	hypothetical protein	MEL_280	9	8
Noumeavirus	NMV_192	hypothetical protein	MEL_234	10	9
Noumeavirus	NMV_225	thioredoxin	MEL_207	11	12
Noumeavirus	NMV_230	hypothetical protein	MEL_202	12	14
Noumeavirus	NMV_045	hypothetical protein	MEL_342b	13	2
Noumeavirus	NMV_238	hypothetical protein	MEL_196	14	24
Noumeavirus	NMV_066	hypothetical protein	MEL_358	15	11
Noumeavirus	NMV_349	hypothetical protein	MEL_065	16	17
Noumeavirus	NMV_163	hypothetical protein	MEL_260	17	15
Noumeavirus	NMV_198	membrane protein	MEL_223b	18	45
Noumeavirus	NMV_068	AAA-familyATPase	MEL_360	19	26
Noumeavirus	NMV_296	hypothetical protein	MEL_097	20	19
Noumeavirus	NMV_058	galactose binding lectin domain containing protein	MEL_352	21	35
Noumeavirus	NMV_060	Transmembrane domain containing protein	MEL_354	22	22
Noumeavirus	NMV_183	membrane protein	MEL_244	23	27
Noumeavirus	NMV_350	hypothetical protein	MEL_061	24	57

Noumeavirus	NMV_355	Transmembrane domain containing protein	N/A	25	N/A
Noumeavirus	NMV_244	ATP-dependenthelicase	MEL_181	26	21
Noumeavirus	NMV_128	thioredoxin	MEL_294	27	25
Noumeavirus	NMV_093	hypothetical protein	MEL_331	28	34
Noumeavirus	NMV_204	hypothetical protein	MEL_231	29	44
Noumeavirus	NMV_074	hypothetical protein	MEL_365b	30	23
Noumeavirus	NMV_248	hypothetical protein	MEL_185	31	28
Noumeavirus	NMV_439	hypothetical protein	MEL_020	32	13
Noumeavirus	NMV_232	Transmembrane domain containing protein	MEL_200	33	16
Noumeavirus	NMV_138	Transmembrane domain containing protein	MEL_282b	34	20
Noumeavirus	NMV_157	Transmembrane domain containing protein	MEL_269	35	30
Noumeavirus	NMV_148	hypothetical protein	MEL_275	36	29
Noumeavirus	NMV_419	histone H2A domain-containing protein	MEL_149	37	38
Noumeavirus	NMV_196	Transmembrane domain containing protein	MEL_228	38	31
Noumeavirus	NMV_218	hypothetical protein	MEL_213b	39	47
Noumeavirus	NMV_221	Transmembrane domain containing protein	MEL_211	40	36
Noumeavirus	NMV_081	hypothetical protein	MEL_371	41	61
Noumeavirus	NMV_441	hypothetical protein	N/A	42	N/A
Noumeavirus	NMV_004	peptidase	MEL_025	43	18
Noumeavirus	NMV_014	peptidase	MEL_041	44	33
A. castellanii	g1525-75307-76608	Euk Porin protein		45	65
Noumeavirus	NMV_214	helicase	MEL_216	46	40
Noumeavirus	NMV_275	lipase	MEL_168	47	32
Noumeavirus	NMV_367	hypothetical protein	MEL_385b	48	82
Noumeavirus	NMV_080	hypothetical protein	MEL_370	49	76
Noumeavirus	NMV_164	Papain-like cysteine protease	MEL_255	50	49
Noumeavirus	NMV_336	amine oxidase	MEL_085	51	74
Noumeavirus	NMV_032	hypothetical protein	MEL_052	52	55
Noumeavirus	NMV_124	ankyrin repeat-containing protein	MEL_292	53	63
Noumeavirus	NMV_166	hypothetical protein	MEL_256	54	67

Noumeavirus	NMV_120	Transmembrane domain containing protein	MEL_301	55	43
Noumeavirus	NMV_212	RibonucleaseIII	MEL_219	56	51
Noumeavirus	NMV_162	SWIB-domain-containing protein	MEL_261	57	98
Noumeavirus	NMV_144	disulfide oxidoreductase	MEL_278	58	52
Noumeavirus	NMV_038	hypothetical protein	MEL_055	59	54
Noumeavirus	NMV_047	hypothetical protein	MEL_341	60	56
Noumeavirus	NMV_006*	peptidase	MEL_040*	61	59
Noumeavirus	NMV_191	serine/threonine protein kinase	MEL_235	62	41
A. castellanii	gunplaced-2126338-2127783	Actin 1		63	78
Noumeavirus	NMV_173	metallopeptidaseWLM	MEL_249	64	68
Noumeavirus	NMV_424	glycosyltransferase	MEL_146	65	84
Noumeavirus	NMV_365	hypothetical protein	MEL_387	66	50
Noumeavirus	NMV_346	hypothetical protein	MEL_066	67	64
Noumeavirus	NMV_132	hypothetical protein	MEL_297	68	46
Noumeavirus	NMV_246	Transmembrane domain containing protein	MEL_183	69	53
Noumeavirus	NMV_005	peptidase	MEL_024	70	87
Noumeavirus	NMV_181	Transmembrane domain containing protein	MEL_245	71	75
Noumeavirus	NMV_216	hypothetical protein	MEL_215	72	48
Noumeavirus	NMV_206	hypothetical protein	MEL_231c	73	95
Noumeavirus	NMV_177	hypothetical protein	MEL_252	74	88
Noumeavirus	NMV_140	thioredoxin	MEL_281	75	91
Noumeavirus	NMV_119	membrane protein	MEL_300	76	90
Noumeavirus	NMV_136	ankyrin repeat-containing protein	MEL_284	77	70
Noumeavirus	NMV_207	hypothetical protein	MEL_232	78	73
Noumeavirus	NMV_170	Transmembrane domain containing protein	MEL_246	79	77
Noumeavirus	NMV_263	Transmembrane domain containing protein	MEL_173	80	81
Noumeavirus	NMV_210	ubiquitin	MEL_221c	81	112
Noumeavirus	NMV_259	hypothetical protein	MEL_177	82	66
Noumeavirus	NMV_176	membraneprotein	MEL_251b	83	69
Noumeavirus	NMV_147	Transmembrane domain containing protein	MEL_276	84	60

Noumeavirus	NMV_361	glycosyltransferase domain containing protein	MEL_398	85	58
Noumeavirus	NMV_389	dual specificity phosphatase	MEL_109	86	93
Noumeavirus	NMV_092	serine/threonine protein kinase	MEL_380	87	37
Noumeavirus	NMV_037	peptidase	MEL_056	88	62
Noumeavirus	NMV_297	hypothetical protein	MEL_098	89	80
Noumeavirus	NMV_359	hypothetical protein	MEL_397	90	96
Noumeavirus	NMV_139	mRNA capping enzyme	MEL_282	91	79
Noumeavirus	NMV_130	glycosyltransferase	MEL_290	92	86
Noumeavirus	NMV_156	Transmembrane domain containing protein	MEL_268	93	92
Noumeavirus	NMV_226	hypothetical protein	MEL_206	94	72
Noumeavirus	NMV_217	hypothetical protein	MEL_214	95	71
Noumeavirus	NMV_154	Transmembrane domain containing protein	MEL_266	96	102
Noumeavirus	NMV_234	class3 lipase	MEL_199	97	94
Noumeavirus	NMV_083	Transmembrane domain containing protein	MEL_373	98	85
Noumeavirus	NMV_347	hypothetical protein	MEL_63b	99	N/A
Noumeavirus	NMV_042	hypothetical protein	MEL_338	100	42
Noumeavirus	NMV_035	hypothetical protein	MEL_133	101	113
A. castellanii	g817-77076-78510	C8 sterol isomerase		102	107
A. castellanii	g1359-61676-62848	hypothetical protein		103	125
Noumeavirus	NMV_126	inactivated thioredoxin/glutaredoxin	MEL_296	104	89
A. castellanii	g254-27747-28588	hypothetical protein		105	103
Noumeavirus	NMV_182	hypothetical protein	MEL_243	106	N/A
Noumeavirus	NMV_385	hypothetical protein	MEL_096	107	100
Noumeavirus	NMV_180	Transmembrane domain containing protein	MEL_259	108	105
A. castellanii	g726-224146-225044	Wnt-10b		109	117
Noumeavirus	NMV_241	hypothetical protein	MEL_193	110	163
A. castellanii	g611-72236-73493	exported protein putative		111	115
Noumeavirus	NMV_007	peptidase	N/A	112	N/A
A. castellanii	g1185-1867882-1870710	aspartic proteinase		113	147
A. castellanii	g376-450681-452588	peroxidase		114	106

A. castellanii	g254-319025-319938	actophorin		115	N/A
A. castellanii	g626-142077-143389	adenine nucleotide translocator		116	N/A
A. castellanii	g641-748251-749192	C-type lysozyme		117	N/A
A. castellanii	g696-284312-285231	LIM domain containing protein		118	145
Noumeavirus	NMV_249	hypothetical protein	MEL_186	119	146
A. castellanii	g1007-388106-392098	hypothetical protein		120	159
Noumeavirus	NMV_348	hypothetical protein	MEL_064	121	99
Noumeavirus	NMV_115	hypothetical protein	MEL_306	122	138
A. castellanii	g757-590645-592959	C2 domain containing protein		123	127
A. castellanii	g422-496887-497476	fascin subfamily protein		124	119
Noumeavirus	NMV_082	hypothetical protein	MEL_372	125	116
A. castellanii	g935-165792-170359	Peroxidase		126	139
A. castellanii	g414-411909-412468	C2 domain containing protein		127	109
Noumeavirus	NMV_269	EBmodulecontainingprotein	MEL_172	128	143
A. castellanii	g42-42086-43890	Rac-like protein		129	173
A. castellanii	g527-541096-542131	Cyclophylin		130	N/A
Noumeavirus	NMV_388	hypotheticalprotein	MEL_108	131	N/A
A. castellanii	g446-42975-43598	hypothetical protein		132	114
A. castellanii	g726-530322-533705	hypothetical protein		133	N/A
A. castellanii	g763-171894-173621	Rab 1B, Ras oncogene family		134	N/A
A. castellanii	g1369-729197-730236	Ras-like protein 1		135	133
A. castellanii	g253-184876-188554	Hydrolase activity		136	181
Noumeavirus	NMV_085	hypothetical protein	MEL_374	137	97
Noumeavirus	NMV_070	hypothetical protein	MEL_361b	138	N/A
A. castellanii	g640-633777-634798	Rho family small GTPase Rac3		139	N/A
Noumeavirus	NMV_363	Transmembrane domain containing protein	MEL_095b	140	N/A
Noumeavirus	NMV_236	hypothetical protein	MEL_197	141	144
A. castellanii	g631-243047-244467	Rab GTPase		142	168
A. castellanii	g1317-154778-156138	Rho family small GTPase Rac3		143	131
A. castellanii	g185-637578-643589	hypothetical protein		144	167

A. castellanii	g631-28616-31737	PA domain containing protein		145	N/A
A. castellanii	g696-243559-244965	Ras family protein		146	N/A
A. castellanii	g218-68723-70868	Corornin3 putative		147	179
A. castellanii	g631-805994-806720	Chorismate mutase subfamily protein		148	128
A. castellanii	g490-123167-124218	hypothetical protein		149	N/A
A. castellanii	g1007-257770-259328	hypothetical protein		150	180
A. castellanii	g414-40155-41168	hypothetical protein		151	182
Noumeavirus	NMV_161	hypothetical protein	MEL_262	152	130
Noumeavirus	NMV_127	hypothetical protein	MEL_295	153	N/A
A. castellanii	g397-418028-419548	Ras GTPase Rap1b		154	164
A. castellanii	g433-73037-74438	Inosineuridine preferring nucleoside hydrolase family protein		155	N/A
A. castellanii	g39-686-2452	EGF-like domain containing protein		156	N/A
A. castellanii	g254-323982-326008	Cysteine proteinase precursor		157	N/A
A. castellanii	g1406-111173-112358	hypothetical protein		158	175
A. castellanii	g1185-1161386-1166670	Amidohydrolase domain containing protein		159	N/A
A. castellanii	g454-198036-200147	EF-hand containing protein		160	120
A. castellanii	g935-470651-471228	Cystatin putative		161	N/A
A. castellanii	g548-81823-84908	I/LWEQ domain containing protein		162	N/A
A. castellanii	g817-118331-118888	hypothetical protein		163	N/A
A. castellanii	g609-257397-258401	hypothetical protein		164	N/A
A. castellanii	g763-133835-134874	Glycosyl hydrolase family 25 subfamily protein		165	N/A
A. castellanii	g376-325048-327718	PhoPQactivated pathogenicity-related protein-like protein; putative		166	N/A
A. castellanii	gunplaced-1973089-1973871	Copper/zinc superoxide dismutase		167	83
A. castellanii	g593-129866-130530	Sequence specific DNA binding		168	N/A
A. castellanii	g1406-789283-793494	Glutamate carboxypeptidase		169	176
A. castellanii	g1449-175373-177058	Ras subfamily protein		170	N/A
A. castellanii	g726-297776-299069	Cysteine proteinase 3, partial		171	N/A
A. castellanii	g360-448580-450093	ImbE family protein		172	N/A
A. castellanii	g680-132237-134308	Protein kinase C4, partial		173	189

A. castellanii	g726-22077-25022	Alkaline phosphatase family subfamily protein		174	N/A
A. castellanii	g593-240006-240660	Peptidylprolyl isomerase FKB12, putative		175	N/A
Noumeavirus	NMV_387	serine/threonine protein kinase	MEL_107	176	186
Noumeavirus	NMV_223	hypothetical protein	MEL_209	177	N/A
A. castellanii	g631-541984-543885	Peptidase S8 and S53 subtilisin kexin sedolisin, putative		178	N/A
A. castellanii	g531-97521-99028	Actin related protein 2/3 complex, 41kDa subunit		179	178
A. castellanii	g527-461746-463738	Ras GTPase		180	155
A. castellanii	gunplaced-1594092-1595819	Factin-capping protein subunit alpha-2; putative		181	N/A
A. castellanii	g307-171526-174660	Glycosyl hydrolase family 20; catalytic domain containing protein		182	N/A
Noumeavirus	NMV_113	zinc ribbon-containing protein	MEL_309	183	140
A. castellanii	gunplaced-519067-520320	hypothetical protein		184	N/A
A. castellanii	g1295-400157-403348	Fasciclin domain containing protein		185	N/A
Noumeavirus	NMV_335	hypothetical protein	MEL_087	186	N/A
A. castellanii	g376-448106-449758	Peroxidase		187	149
A. castellanii	gunplaced-452590-455256	Ef1a		188	142
A. castellanii	g1295-157226-158056	hypothetical protein		189	N/A
A. castellanii	g284-678893-681196	PH domain containing protein		190	161
A. castellanii	g1317-24676-26730	Prokumamolisin, putative		191	N/A
A. castellanii	cox1/2	cox1/2		192	192
A. castellanii	g535-133406-135131	ATP:L-Methionine S-Adenosyltransferase		193	N/A
A. castellanii	g1185-1806178-1808434	Protein phosphatase 1 catalytic subunit alpha, putative		194	134
A. castellanii	g763-137286-138388	Glycosyl hydrolase family 25 subfamily protein		195	N/A
A. castellanii	g414-25246-26688	Sar1 family small GTPase		196	N/A
A. castellanii	g406-2586-7412	Lysosomal alphamannosidase		197	N/A
A. castellanii	g297-276196-278530	Prokumamolisin, activation domain containing protein		198	N/A
A. castellanii	g536-73396-74613	hypothetical protein		199	N/A
A. castellanii	g1196-29473-31845	amineoxidase, Flavine containing superfamily protein		200	N/A
A. castellanii	g185-111308-112608	autophagy-related protein 27		201	135

A. castellanii	g1185-1240877-1242718	PH domain containing protein		202	N/A
Noumeavirus	NMV_242	hypothetical protein	MEL_192	203	185
A. castellanii	g1390-216047-218303	High molecular weight heat shock protein		204	191
A. castellanii	g1306-148886-150120	Vacuolar proton ATPase, putative		205	N/A
A. castellanii	g739-222522-223876	Fibrillarin, putative		206	N/A
A. castellanii	g734-49529-51643	Peptidase M16 family protein		207	N/A
A. castellanii	g1449-118768-120377	Actin related protein 2/3 complex		208	N/A
A. castellanii	g1369-497445-500306	Gelation factor, putative		209	169
A. castellanii	gunplaced-2251673-2253071	ARP2/3 complex 34 kDa subunit, putative		210	N/A
Noumeavirus	NMV_400	zinc finger protein	MEL_347	211	152
A. castellanii	g254-213866-215830	Peptidase M16 inactive domain containing protein		212	188
A. castellanii	g1185-98157-99558	Histone cluster 1, H2bb, putative		213	158
A. castellanii	gunplaced-1669823-1671094	Mn/Fe superoxide dismutase		214	166
A. castellanii	g1185-1855544-1856934	Mitochondrial phosphate transporter, putative		215	N/A
A. castellanii	g631-162107-163748	Tubulin beta chain		216	141
A. castellanii	g527-210936-212510	Ras-related protein Rab-21		217	N/A
A. castellanii	g171-459415-462379	amino acid transmembrane transport		218	N/A
Noumeavirus	NMV_229	hypothetical protein	MEL_203	219	N/A
A. castellanii	g360-68713-70325	RNA recognition motif domain containing protein		220	N/A
A. castellanii	g433-288-2349	Carbohydrate metabolic process		221	N/A
A. castellanii	g185-569957-572411	phospholipid binding		222	N/A
A. castellanii	g544-146276-147708	Factin-capping protein subunit beta; putative		223	N/A
A. castellanii	g509-307446-310070	Serine carboxypeptidase (CPB1), putative		224	N/A
A. castellanii	g677-37001-39098	Actin related protein 3, putative		225	N/A
A. castellanii	g531-32516-35280	V ATPase subunit A, putative		226	N/A
A. castellanii	g171-371925-373194	RSNARE, VAMP71-family		227	N/A
A. castellanii	g935-641463-642298	Cytochrome C Oxydase		228	N/A
A. castellanii	g1185-112487-113838	Glyceraldehyde-3-phosphate dehydrogenase, cytosolic-like		229	172
A. castellanii	g1390-714707-716810	Alpha tubulin, partial		230	132

A. castellanii	g993-461397-463268	Protein P80, putative		231	N/A
A. castellanii	g1007-410536-413510	Predicted protein		232	170
A. castellanii	g452-22276-25291	ATP synthase, putative		233	N/A
A. castellanii	g218-115547-116688	Peroxiredoxin 2, putative		234	N/A
A. castellanii	g993-399293-400878	RAB11B protein, putative		235	N/A
Noumeavirus	NMV_075	hypothetical protein	MEL_366	236	N/A
Noumeavirus	NMV_312	ATPase	MEL_141	237	150
A. castellanii	g757-61267-67282	Amidohydrolase domain containing protein		238	N/A
A. castellanii	g481-83586-84700	Actin related protein ARPC3, putative		239	N/A
A. castellanii	g757-460169-461398	START domain containing protein		240	N/A
A. castellanii	g327-32846-34973	Malate dehydrogenase		241	N/A
A. castellanii	g490-271761-274518	Eukaryotic translation elongation factor 2, putative		242	N/A
A. castellanii	g397-224837-226378	Rab7 GTPase		243	N/A
A. castellanii	g1067-140826-143107	Glycoside hydrolase family protein		244	N/A
A. castellanii	g1185-686457-688604	Cell cycle control protein		245	N/A
A. castellanii	g1271-599999-602983	Acyloxyacyl hydrolase		246	N/A
A. castellanii	g527-633449-637592	Cobalaminin dependent methionine synthase		247	195
A. castellanii	g1317-556358-557497	protein domain specific binding		248	177
A. castellanii	g171-452759-454367	Rab4, putative		249	N/A
A. castellanii	g935-656616-658717	Plastin 3 (T isoform), putative		250	N/A
A. castellanii	g1377-88396-89795	Synaptobrevin protein		251	N/A
A. castellanii	g527-274667-278548	Gamaglutamyl transferase		252	N/A
A. castellanii	g448-516809-518949	Coenzyme binding		253	N/A
A. castellanii	g376-14236-15730	hypothetical protein		254	N/A
A. castellanii	g1359-44666-49600	Oxidation-reduction process		255	N/A
A. castellanii	g1369-642385-649286	P53 inducible protein		256	N/A
A. castellanii	g1007-235917-237588	Soluble NSF attachment protein alpha isoform		257	N/A
A. castellanii	g557-252166-256085	Glycosyl hydrolase family 65 central catalytic domain containing protein		258	N/A
A. castellanii	g422-858104-863968	Amidohydrolase domain containing protein		259	N/A

A. castellanii	g1317-272306-275600	Sec1 family protein, partial		260	190
A. castellanii	g527-226157-232618	Myosin-I binding protein Acan125		261	N/A
A. castellanii	atp1	ATP11 protein		262	N/A
A. castellanii	g535-204667-205958	Ras family GTPase		263	N/A
A. castellanii	g40-21764-24998	BnaC01g40190D		264	N/A
A. castellanii	gunplaced-469113-471317	Ribosomal protein S32, putative		265	N/A
A. castellanii	g40-189273-197081	RecName: Full		266	N/A
A. castellanii	g311-83679-90488	Phospholipid translocating P-type ATPase family protein		267	N/A
A. castellanii	g1185-932177-934308	Vacuolar ATPase subunit DVA41		268	N/A
A. castellanii	g185-395816-398240	hypothetical protein		269	N/A
A. castellanii	g904-22075-24548	H(+)-transporting ATPase family protein		270	N/A
A. castellanii	g641-620276-623240	Zinc finger, C3HC4 type (RING finger) domain containing protein		271	N/A
A. castellanii	g185-371587-375028	Agarase, putative		272	N/A
A. castellanii	gunplaced-225545-227583	hypothetical protein		273	N/A
A. castellanii	g626-155026-157500	Chaperonin GroL, putative		274	N/A
Noumeavirus	NMV_356	ATP-dependent DNA ligase	MEL_060	275	187
A. castellanii	g734-24891-27760	Tripeptidylpeptidase 1, putative		276	N/A
A. castellanii	g631-1049155-1053916	Protein binding		277	N/A
Noumeavirus	NMV_129	hypothetical protein	MEL_293	278	N/A
A. castellanii	g1297-265817-271878	Myosin-IA		279	N/A
A. castellanii	g430-68666-70450	Ras subfamily protein, putative		280	N/A
A. castellanii	g1229-124396-134855	Binding		281	N/A
A. castellanii	g218-42810-52640	GTPase-activator protein for Ras family GTPase		282	N/A
A. castellanii	g1185-1772532-1781891	Multidrug resistance associated protein, putative		283	N/A
A. castellanii	g1185-770357-772028	Multidrug resistance associated protein, putative		N/A	101
A. castellanii	g557-519644-520579	Vtype ATPase; C subunit		N/A	104
A. castellanii	g935-64777-65458	Ribosomal L40e family		N/A	108
Noumeavirus	NMV_401	hypothetical protein	MEL_348	N/A	110
	N/A	Transmembranedomaincontainingprotein	MEL_332	N/A	111

Noumeavirus	NMV_061	uracil-DNAglycosylase	MEL_355	N/A	118
Noumeavirus	NMV_301	hypothetical protein	MEL_102	N/A	121
A. castellanii	g97-87038-122304	Probable aggregation factor core protein MAFp3		N/A	122
A. castellanii	g97-85422-86868	hypothetical protein		N/A	123
	N/A	hypothetical protein	MEL_129b	N/A	124
Noumeavirus	NMV_231	Hypothetical protein	MEL_201	N/A	126
Noumeavirus	NMV_123	Zinc finger protein	MEL_286	N/A	129
Noumeavirus	NMV_122	hypothetical protein	MEL_287	N/A	136
	N/A	endonuclease	MEL_221b	N/A	137
	N/A	hypothetical protein	MEL_323b	N/A	148
A. castellanii	g284-245837-247148	hypothetical protein		N/A	151
Noumeavirus	NMV_188	hypothetical protein	MEL_238	N/A	153
Noumeavirus	NMV_360	hypothetical protein	MEL_049	N/A	154
A. castellanii	g40-197330-199538	Tubulin beta1 chain		N/A	156
A. castellanii	g422-400365-406076	Ubiquitin carboxylterminal hydrolase domain containing protein		N/A	157
Noumeavirus	NMV_152	eukaryotic translation initiation factor 5	MEL_273	N/A	160
Noumeavirus	NMV_260	hypothetical protein	MEL_176	N/A	162
	N/A	adenine-specific methyltransferase	MEL_016	N/A	165
A. castellanii	g1369-335797-338290	Calponin domain containing protein		N/A	171
Noumeavirus	NMV_418	hypothetical protein	MEL_171	N/A	174
Noumeavirus	NMV_067	helicase	MEL_359	N/A	183
Noumeavirus	NMV_253	proliferating cell nuclear antigen	MEL_190	N/A	184
A. castellanii	g1271-609961-613385	Pigment precursor permease subfamily protein		N/A	193
A. castellanii	gunplaced-1642808-1647994	Protein kinase domain containing protein		N/A	194
A. castellanii	g397-60057-61818	Hypothetical protein		N/A	196

* NMV_006 and MEL_040 are non orthologous homologues

Supplementary Table 2: Host proteins shared by the Noumeavirus and Melbournevirus virions and their ranks in virions and non-infected *A. castellanii* cells (NI)

Gene	Protein name	Real rank in Noumea virus	Real rank in Melbourne virus	Host relative rank in Noumea virus	Host relative rank in Melbourne virus	Rank in host (NI)	Other viruses
**gunplaced-2126338-2127783**	Actin 1	63	78	2	2	1	+
**g422-496887-497476**	fascin subfamily protein	124	119	13	11	2	+
**g1525-75307-76608**	Euk Porin protein	45	65	1	1	3	+
**g1185-112487-113838**	Glyceraldehyde-3-phosphate dehydrogenase, cytosolic-like	229	172	47	37	4	0
**g414-411909-412468**	C2 domain containing protein	127	109	15	7	5	+
**g696-284312-285231**	LIM domain containing protein	118	145	10	24	9	+
**g527-633449-637592**	Cobalaminin dependent methionine synthase	247	195	50	52	10	0
**g1390-216047-218303**	High molecular weight heat shock protein	204	191	41	50	12	+
**g1317-556358-557497**	Hypothetical, protein binding	248	177	51	41	18	0
**g218-68723-70868**	Corornin3 putative	147	179	23	43	42	+
**gunplaced-1973089-1973871**	Copper/zinc superoxide dismutase	167	83	30	3	54	+
**g254-213866-215830**	Peptidase M16 inactive domain containing protein	212	188	43	47	72	+
**g726-224146-225044**	Wnt-10b	109	117	6	10	92	+
**g1359-61676-62848**	hypothetical protein	103	125	4	13	129	+
**g757-590645-592959**	C2 domain containing protein	123	127	12	14	138	+
**g1390-714707-716810**	Alpha tubulin, partial	230	132	48	17	140	0
**g531-97521-99028**	Actin related protein 2/3 complex, 41kDa subunit	179	178	33	42	152	+
**g631-162107-163748**	Tubulin beta chain	216	141	46	22	164	0
**g376-450681-452588**	peroxidase	114	106	9	5	174	+
**gunplaced-1669823-1671094**	Mn/Fe superoxide dismutase	214	166	45	32	188	0
**cox1/2**	cox1/2	192	192	38	51	220	+
**g1406-111173-112358**	hypothetical protein	158	175	28	39	224	0
**g42-42086-43890**	Rac-like protein	129	173	16	38	269	0
**g446-42975-43598**	hypothetical protein	132	114	17	8	286	+
**g1369-497445-500306**	Gelation factor, putative	209	169	42	35	290	0
**g284-678893-681196**	PH domain containing protein	190	161	37	30	308	+
**g376-448106-449758**	Peroxidase	187	149	35	26	309	+
**g1369-729197-730236**	Ras-like protein 1	135	133	18	18	331	0
**g817-77076-78510**	C8 sterol isomerase	102	107	3	6	333	+
**g611-72236-73493**	exported protein putative	111	115	7	9	338	0
**g631-243047-244467**	Rab GTPase	142	168	20	34	356	0
**g185-111308-112608**	autophagy-related protein 27	201	135	40	20	370	+
**g397-418028-419548**	Ras GTPase Rap1b	154	164	27	31	374	0
**g1317-154778-156138**	Rho family small GTPase Rac3	143	131	21	16	401	0
**g1185-1867882-1870710**	aspartic proteinase	113	147	8	25	475	+
**g414-40155-41168**	hypothetical protein	151	182	26	46	484	0
**g527-461746-463738**	Ras GTPase	180	155	34	27	487	+
**g680-132237-134308**	Protein kinase C4, partial	173	189	32	48	521	0
**g454-198036-200147**	EF-hand containing protein	160	120	29	12	524	+
**g1007-410536-413510**	Predicted protein	232	170	49	36	570	+
**g935-165792-170359**	Peroxidase	126	139	14	21	789	+
**g1185-1806178-1808434**	Protein phosphatase 1 catalytic subunit alpha, putative	194	134	39	19	837	+
**g1007-257770-259328**	hypotheticalprotein	150	180	25	44	865	+
**g254-27747-28588**	hypothetical protein	105	103	5	4	1080	+
**g1317-272306-275600**	Sec1 family protein, partial	260	190	52	49	1143	+
**g1007-388106-392098**	hypothetical protein	120	159	11	29	1391	0
**g253-184876-188554**	Hydrolase activity	136	181	19	45	1555	+

**g1185-98157-99558**	Histone cluster 1, H2bb, putative	213	158	44	28	1621	0
**g1406-789283-793494**	Glutamate carboxypeptidase	169	176	31	40	1783	0
**g631-805994-806720**	g1406-111173-112358	148	128	24	15	2601	+
**g185-637578-643589**	hypothetical protein	144	167	22	33	3020	+
**gunplaced-452590-455256**	Ef1a	188	142	36	23	NA	0

Supplementary Table 3: Identification and ranking of proteins involved in transcription and transcript maturation in the virion proteomes of cytoplasmic viruses *versus* Noumeavirus and Melbournevirus

Gene name	Mimivirus				Pithovirus sibericum				Noumeavirus				Melbournevirus			
	Gene #	Theore-tical	Identified Peptides	Rank	Gene #	Theore-tical	Identified Peptides	Rank	Gene #	Theore-tical	Identified Peptides	Rank	Gene #	Theore-tical	Identified Peptides	Rank
**Rpb1**	R501	77	50	65	pv_366 -368	51	35	68	NMV_011	70	noID		MEL_047	72	noID	
**Rpb2**	L244	60	30	80	pv_242	61	40	78	NMV_436	55	noID		MEL_022	63	noID	
**Rpb5**	L235	12	7	54	pv_31	11	8	72	NMV_046	14	noID		MEL_342	9	noID	
**polyA pol**	R341	30	14	87	NA	NA	NA	NA	NA	NA	NA	NA	NA	NA	NA	NA
**RNAse III**	R343	28	noID		pv_75	20	15	63	NMV_212	18	10	56	MEL_219	16	8	51
**mRNA capping enzyme**	R382	72	41	83	pv_454	52	38	52	NMV_139	45	28	93	MEL_282	46	28	80

The table presents the number of theoretically identifiable (http://web.expasy.org/peptide_mass) and experimentally identified peptides (no miscleavage allowed, minimum of 7 amino acids, m/z between 750 and 4000 Da) as well as the rank observed for the corresponding genes for Mimivirus, *Pithovirus sibericum*, Noumeavirus and Melbournevirus. NA: the corresponding gene does not exist, noID: no identified peptide

Supplementary Table 4: Conservation of Noumeavirus transcription-related genes in Marseilleviridae and *A. castellanii*

	PAP	RPB1	RPB2	RPB5	RPB9
**Canne 8 virus**	Not present	65%	69%	52%	69%
**Melbournevirus**	Not present	68%	69%	52%	69%
**Marseillevirus**	Not present	67%	69%	52%	68%
**Tokyovirus**	Not present	65%	70%	53%	69%
**Port-miou virus**	Not present	93%	94%	92%	94%
**Laussanevirus**	Not present	93%	94%	92%	94%
**Noumeavirus**	Not present	100%	100%	100%	100%
**Insectomimevirus**	Not present	78%	77%	74%	90%
**Tunisvirus**	Not present	78%	77%	73%	90%
**Brazilian marseillevirus**	Not present	79%	76%	73%	94%
**Golden mussel marseillevirus**	Not present	66%	66%	59%	Not present (frameshifted)
A. castellanii	NA	29%	32%	25%	18%

The sequence identity at the protein level was calculated relative to Noumeavirus. *A. castellanii* and Noumeavirus RPB9 were too divergent to get a reliable phylogenetic reconstruction. This gene was not included in Supplementary Fig. 2.

Supplementary Table 5: Specific primers used for RT-PCR

NMV_1_RT-F	CTT CCT TGA GGA ACA CTT CAA G
NMV_11_RT-F	TAT CTT CCC ATT GAT GTG CTC
NMV_11_RT-R	GAC TCT CTG ATA ACC TTG CAC ACC
NMV_125_RT-F	CTC AAG TTC GAT TGG AAC TAT C
NMV_125_RT-R	TCA TAA TAT CCT GTC TTC CCG
NMV_252_RT-F	CTA CGG TTC GGA ATT TGA G
NMV_252_RT-R	TCT TGA TTT TGG GAA TTT CC
NMV_436_RT-F	AAG TCG GAG ACA AGG CGA C
NMV_436_RT-R	GTC GAG TAA CGA ATC GCA AC
NMV_287_RT-F	TCG TGA CTT ATA GAA GGT CAC
NMV_288_RT-R	GCC ATG ATG TAT TTC TTG ATG
NMV_189_RT-F	ATG TCA GTA TAC GGA CCT GTC CCC
ACpap-RT_F	AGG ACA CTC CGA ACG TGA AGT AC
ACpap-RT_R	GTT TCC ATC ATC ATC TCC CTC G
ACrpb1-RT_F	GAA GGG ACT GGT CAA GGT GCT G

ACrpb1-RT_R	CAT GAG AGT GAG GTT GTT CCA GGG
Mel_47-RT_F	CGT TTT GAC AGT GAG GAA GAT G
Mel_47-RT_R	AAC GAA AGA GAT GTT GTC GCT C
Mel_22_RT-F	GAA CTC TGT CAG CAA GAA TTC
Mel_342_RT-F	AAC TTT CGA GAG CGA AGA AG
Mel_305_RT-F	GTT TCT TAC GAC TCG ACT TTC
Mel_236_RT-F	TCA GGA TTC GGT CCC ATC C

Supplementary Methods

Phylogenetic analyses

Noumeavirus was positioned within the Marseilleviridae family using the orthologous DNA polymerase protein (DNApol) sequences from Brazilian marseillevirus (YP_009238893), Cannes 8 virus (AGV01694), Lausannevirus (YP_004347308), Insectomime virus (AHA45970), Melbournevirus (AIT54904), Port-Miou virus (ALH07009), Tunisvirus fontaine2 (AHC54969) and Tokyovirus (NC_030230). Since the DNApol sequences of Marseillevirus (NC_013756) and Golden mussel marseillevirus (NC_031465.1) were not annotated, they were recovered using the "protein2genome" option of Exonerate[3]. The Iridovirus Invertebrate iridescent virus 3 (YP_654692) was used as an outgroup. We then performed a multiple alignment using Mafft[4] with the L-INS-i parameter. The best model for phylogenetic tree reconstruction was chosen using Protest[5] (i.e. LG+G) and RaxML[1] was finally used to build the tree. We next used OrthoMCL[6] with standard parameters and predicted proteins with a sequence length > 100 amino acids to generate clusters of Marseilleviridae homologs. We retained clusters containing 1:1 orthologs of each virus (n=21), aligned them, concatenated the alignments and computed the tree as for the above phylogenetic tree.

RNA extraction, mRNA enrichment and RT-PCR of virion-associated mRNAs

To investigate whether mRNAs were packaged in the viral particles, we extracted RNA from purified virions and used Noumeavirus- and Melbournevirus-infected *A. castellanii* cells as a positive control. Three different quantities of Noumeavirus and Melbournevirus purified particles (5.10^9, 5.10^{10} and 5.10^{11}) were treated with 20µg mL^{-1} DNase-free RNase (Life Technologies) for 10 minutes at room temperature. The viral pellets were washed 3 times in PBS and incubated with 1U μL^{-1} of RNasin ribonuclease inhibitor (Promega) for one hour at 37°C. After 3 additional washes in PBS, the pellets were resuspended in RLT buffer supplemented with B-mercaptoethanol. For the positive controls, infections with Noumeavirus and Melbournevirus were performed in 7 T25 flasks containing 100 000

A. castellanii cells per cm^2 at MOI 50. The flasks were collected every hour from 1h to 7h pi. All cell pellets were pooled and resuspended in RLT buffer supplemented with B-mercaptoethanol.
RNAs were extracted using the RNeasy mini kit (Qiagen) according to the manufacturer protocol. One step of RNase-free DNase digestion was performed on-column using the RNase-Free DNase Set (Qiagen) and total RNAs were eluted in RNase-free water. Except for 4µL, total RNAs were used for mRNA enrichment (Life Technologies, Dynabeads oligodT25). The 4 µL total RNAs and purified mRNAs were reverse transcribed using an oligodT(25) primer and the SMARTScribe Reverse Transcriptase (Clontech Laboratories) and then treated with RNase H. Noumeavirus and Melbourne virions were tested for the presence of viral (NMV_011 and MEL_047) and cellular RPB1 (ACrpb1) transcripts, for the RPB2 subunit of Melbournevirus (MEL_022), for the transcripts of the most abundant proteins in the virions (NMV_189 and MEL_236), the major capsid protein (MEL_305) and for the cellular polyA polymerase (ACpap) mRNA (Supplementary Table 5). PCR products were analyzed on 1.2% agarose gel and sent out for sequencing.
To estimate whether the amplicons corresponding to the RPB1 mRNA in Melbournevirus virions were compatible with one molecule of mRNA per particle, we added a quantity of commercial GFP mRNAs (OZ Biosciences) corresponding to one molecule per particle in the lysates of 10^9, 5.10^9 and 5.10^{10} virions. We then performed the mRNA purification, reverse transcription and PCR amplification as described above.

Supplementary Note 1

After RNA purification from $5x10^9$ to $5x10^{11}$ particles of Noumeavirus and Melbournevirus, the RNA was quantified using the Qubit fluorometric quantification (Thermofisher). The corresponding signal was much lower than the DNA signal (3 to 10 times) despite multiple treatments with DNAse. As the viruses RPB1 genes contain a self-splicing intron and the host RPB1 contains 24 introns, we focused on their transcripts to investigate the presence of mRNA corresponding to the virus and host transcription machineries in the two Marseilleviridae virions. No amplicons was obtained for the host RPB1 and PAP mRNAs on total RNA and mRNA extracted from the two virions, while amplicons were readily obtained from the infected cells. Amplicons of the virus-encoded RPB1 mRNA were obtained in infected cells and from the mRNA extracted from Melbournevirus when using $>10^{10}$ virions (Supplementary Fig. 1). In contrast, none was obtained with Noumeavirus or when using $5x10^9$ Melbournevirus virions. The mRNA corresponding to the virus-encoded RPB2 was not detected in Melbournevirus particles. As a control for possible bystanders, the mRNA of the most abundant protein (NMV_189 and MEL_236) was detected in both virions, while the mRNA of the Major Capsid Protein was only detected in Melbournevirus, suggesting that, as for the RPB1 mRNA, they most

likely corresponded to bystanders at less than one copy per particle (Supplementary Fig. 1). This was confirmed by the use of commercial GFP mRNA which produced an intense band on agarose gel revealed by BET, with 1 molecule per virion even when using 10^9 particles, a quantity for which RPB1 was never amplified from Noumeavirus or Melbournevirus particles (Supplementary Fig. 1). This argues for the bystander nature of the mRNAs identified in the virions.

Supplementary References

1. Stamatakis, A. RAxML version 8: a tool for phylogenetic analysis and post-analysis of large phylogenies. *Bioinforma. Oxf. Engl.* **30,** 1312–1313 (2014).
2. Krupovic, M. & Koonin, E. V. Self-synthesizing transposons: unexpected key players in the evolution of viruses and defense systems. *Curr. Opin. Microbiol.* **31,** 25–33 (2016).
3. Slater, G. S. C. & Birney, E. Automated generation of heuristics for biological sequence comparison. *BMC Bioinformatics* **6,** 31 (2005).
4. Katoh, K. & Standley, D. M. MAFFT multiple sequence alignment software version 7: improvements in performance and usability. *Mol. Biol. Evol.* **30,** 772–780 (2013).
5. Darriba, D., Taboada, G. L., Doallo, R. & Posada, D. ProtTest 3: fast selection of best-fit models of protein evolution. *Bioinformatics* **27,** 1164–1165 (2011).
6. Li, L., Stoeckert, C. J. & Roos, D. S. OrthoMCL: Identification of Ortholog Groups for Eukaryotic Genomes. *Genome Res.* **13,** 2178–2189 (2003).
